# Lung Mechanics Over the Century: From Bench to Bedside and Back to Bench

**DOI:** 10.3389/fphys.2022.817263

**Published:** 2022-07-13

**Authors:** Paolo Jose Cesare Biselli, Fernanda Degobbi Tenorio Quirino Dos Santos Lopes, Renato Fraga Righetti, Henrique Takachi Moriya, Iolanda Fátima Lopes Calvo Tibério, Milton Arruda Martins

**Affiliations:** ^1^ Intensive Care Unit, University Hospital, University of Sao Paulo, Sao Paulo, Brazil; ^2^ Laboratory of Experimental Therapeutics, Department of Clinical Medicine, School of Medicine, University of Sao Paulo, Sao Paulo, Brazil; ^3^ Hospital Sírio-Libanês, Serviço de Reabilitação, São Paulo, Brazil; ^4^ Biomedical Engineering Laboratory, Escola Politecnica, University of Sao Paulo, Sao Paulo, Brazil

**Keywords:** lung mechanics modelling, equation of motion, constant-phase model, respiratory diseases, animal models—rodent, critical care, mechanical ventilalion, lung physiology

## Abstract

Lung physiology research advanced significantly over the last 100 years. Respiratory mechanics applied to animal models of lung disease extended the knowledge of the workings of respiratory system. In human research, a better understanding of respiratory mechanics has contributed to development of mechanical ventilators. In this review, we explore the use of respiratory mechanics in basic science to investigate asthma and chronic obstructive pulmonary disease (COPD). We also discuss the use of lung mechanics in clinical care and its role on the development of modern mechanical ventilators. Additionally, we analyse some bench-developed technologies that are not in widespread use in the present but can become part of the clinical arsenal in the future. Finally, we explore some of the difficult questions that intensive care doctors still face when managing respiratory failure. Bringing back these questions to bench can help to solve them. Interaction between basic and translational science and human subject investigation can be very rewarding, as in the conceptualization of “Lung Protective Ventilation” principles. We expect this interaction to expand further generating new treatments and managing strategies for patients with respiratory disease.

## Introduction

Respiratory mechanics has been extensively studied during the last century (RAHN et al., 1946; Otis, 1977; Collett et al., 1985; Bates, 2005) with resultant improvement in our understanding of the function of the respiratory system in health and disease states (MEAD et al., 1955; Reinert and Trendelenburg, 1972; Dodd et al., 1988; Mador, 1991). Newfound knowledge of respiratory mechanics has also been applied to different animal models of respiratory disease (Wanner and Abraham, 1982; Wanner et al., 1990; Irvin and Bates, 2003).

Over the years, accumulated knowledge in respiratory mechanics has been incorporated into mechanical ventilators and respiratory functional assessment of patients (Younes, 1992; Sinderby et al., 1999; Jonkman et al., 2020). Respiratory mechanics became not only a tool for investigating lung disorders. It was also used in developing treatments for failing respiratory system and in designing strategies to prevent lung injury (Henderson et al., 2017).

In this review, we describe some of the mathematical approaches used in respiratory mechanics in animal models and in patients and their application in the development of mechanical ventilation. Finally, we will discuss how respiratory mechanics is still important for both research and clinical care and how it can provide insightful information moving respiratory science forward.

## Respiratory Mechanics in Animal Models

Animal models have been extensively used to elucidate different physiological mechanisms leading to respiratory disease development, such as asthma and chronic obstructive pulmonary disease (COPD).

Generally, the assessment of respiratory mechanics in animal models is based on the acquisition of pressure and volume/flow data. From these data, mathematical models whose parameters have physiological significance are applied (Bates, 2005).

**FIGURE 1:**
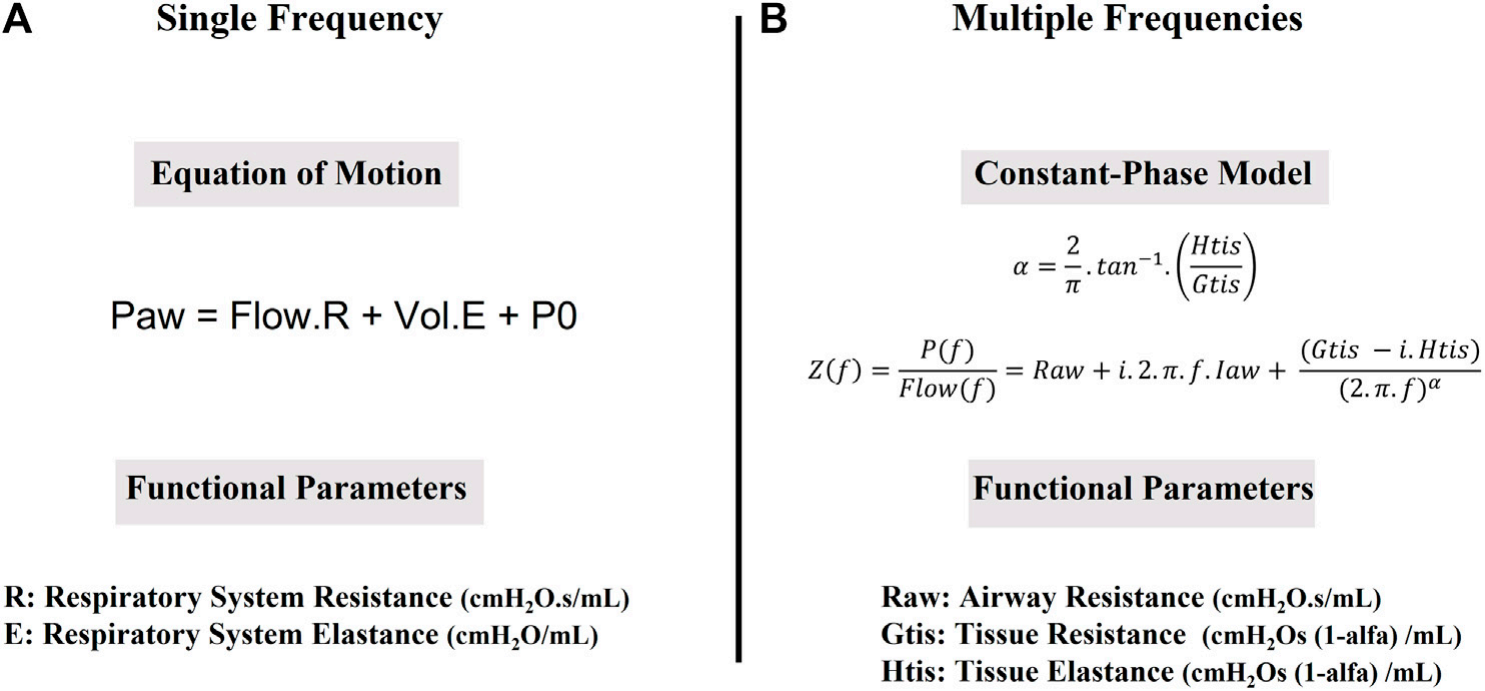
Mathematical models used to obtain functional parameters. **(A)**. The equation of Motion: Paw (cmH_2_O) is airway pressure, measured at airway opening, Flow (mL/s) is airway flow, R is Respiratory System Resistance (cmH_2_O.s/mL), Vol (mL) is the Volume of air that has entered the lungs since the beginning of inspiration, E (cmH_2_O/mL) is Respiratory System Elastance and P0 (cmH_2_O) is the airway pressure at the beginning of inspiration. **(B)**. The Constant Phase Model (Z(f), cmH_2_O.s/mL) is calculated as the pressure response (P(f), cmH_2_O) divided by ventilator generated flow (Flow(f), mL/s) at each frequency (f). Raw represents Newtonian resistance (cmH_2_O.s/mL); i is imaginary number; Iaw is airway inertance (cmH_2_O.s^2^/mL), Gtis is tissue viscance (cm H_2_O.s^(1−α^)/mL); Htis is tissue elastance (cm H_2_O.s^(1−α)^/mL).

The most used model for evaluating respiratory mechanics is known as the “equation of motion.” It is a linear one-compartment model that assumes the respiratory system is excited at a single frequency, usually very close to the respiratory rate (Figure 1) (Bates, 2005). The parameters of this model are respiratory system resistance, representing the amount of pressure required to generate flows; and respiratory system elastance, which is the amount of pressure required to maintain volume changes in the respiratory system.

**FIGURE 2:**
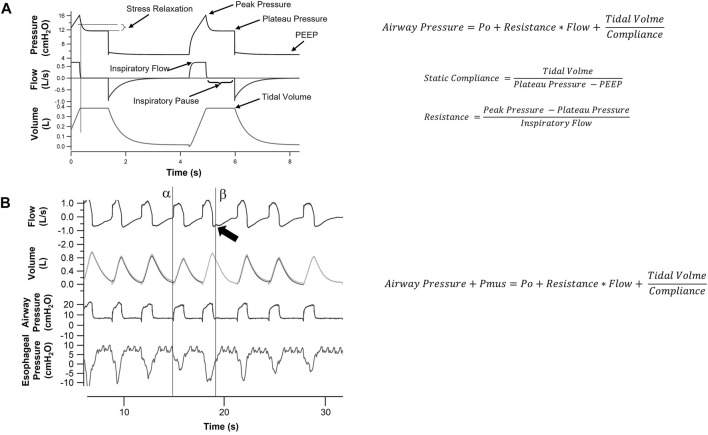
Respiratory mechanics in paralyzed and spontaneously breathing patients. **(A)**. This is a representation of Flow, Volume and Pressure signals over time for a patient under sedation and neuromuscular blockage. Since there is no respiratory muscle activity, equation of motion (see Figure 1A) can be applied. Compliance (inverse of Elastance, displayed in Figure 1) and Resistance can be found using minimum square method or by the inspiratory pause special case displayed in **(A)**. Applying an inspiratory pause will simplify the equation allowing measurement of Static Compliance and Resistance. It also allows observation of viscoelastic properties of the respiratory system, with the slow decrease in pressure (Stress Relaxation) once flow is abruptly stopped. **(B)**. In non-sedated patients, respiratory muscles interact with the ventilator. In this example, we show Pressure, Flow, Volume and Esophageal Pressure signal over time in a patient ventilated in Pressure Support Mode. Muscle activity can be measured with an esophageal catheter. In this scenario, equation of motion needs to be adapted to include pressure generated by respiratory muscles (Pmus). The model becomes less stable but esophageal pressure reveals several features of patient ventilator interaction. The observed changes in tidal volumes over several breaths is caused by a change in patient’s effort detected by esophageal pressure. Additionally, we can observe the inspiratory effort triggering mechanical ventilator (line α) and the prolongation of patient’s inspiratory effort into ventilator expiration (line β), distorting expiratory flow curve (arrow).

The equation of motion does not contemplate viscoelastic phenomenon. Viscoelasticity is the property to accommodate stress following changes in volume (stress relaxation) (Faffe and Zin, 2009). It can be observed in pressure-time graph once inspiratory flow is abruptly stopped. The slow pressure decrease after inspiratory pause reveals stress relaxation (Figure 2A). It results from parenchymal fiber conformational adaptation (Faffe and Zin, 2009), changes in surface tension in water-air interfaces or redistribution of air within lung regions (Bates, 2009). Measurements in viscoelasticity add complexity to equation of motion model.

The respiratory system has a high frequency dependence. This led to the use of excitations with more than one frequency in order to calculate the respiratory impedance that basically characterizes the biomechanical behaviour of the respiratory system in the frequencies contained in the excitation (Figure 1) (Hantos et al., 1992).

For a better physiological understanding of respiratory impedance, models are used whose parameters are correlated with dissipative (airway resistance and tissue viscosity) and conservative (elastance or tissue compliance) components. The most used model for understanding respiratory impedance in small rodents is the constant-phase model (Bates and Irvin, 2003). This model provides more detailed information on lung mechanics comparing to equation of motion. It describes proximal and distal airways with different parameters, which can be useful to evaluate obstructive diseases.

### Respiratory Mechanics in Animal Models of Lung Disease

Respiratory mechanics have been studied in several animal models of respiratory diseases. In experimental emphysema, researchers observed a decrease in tissue elastance and viscosity (see section “Respiratory mechanics in animal models”) related to alveolar destruction and loss of viscoelastic properties (Bates, 2009; Ito et al., 2019a, 2019b). On the other hand, repairing lung tissue with different components of extracellular matrix also alters lung function. Changes in collagen fibers types I and III and elastin in lung parenchyma leads to loss of lung elasticity (Fietzek and Kuehn, 1976; Shifren et al., 2007; Suki and Bates, 2008; Koenders, 2009).

Airway resistance increase is usually detected in experimental models of asthma where structural changes in airways are the major histological finding (Bates, 2009). Edema in peribronchovascular areas also increases viscoelastic properties. Airway hyperresponsiveness can be detected with a dose-response curve to methacholine or the antigen itself (Wang et al., 1986). Both constant phase model and equation of motion can be used to describe respiratory function in these animals (Bates, 2009) (see Figure 1 and section “Respiratory mechanics in animal models” above).

Camargo et al. (2020) showed in an experimental model of asthma in mice sensitized with ovalbumin that there was an increased response of airway resistance when compared to the control group. In addition, Possa et al. (2012); Righetti et al. (2014) and Pigati et al. (2015), in an experimental model of asthma showed that the worsening in mechanical parameters had a positive correlation with markers of eosinophilic inflammation, Th2 profile cytokines, oxidative stress (iNOS and 8- iso-PGF2alpha), and extracellular matrix remodelling.

Oscillatory mechanics and the constant phase model (see Figure 1 and section “Respiratory mechanics in animal models” above) can be applied not only to whole lung, but also to lung tissue *in vitro*. Using this technique, it is possible to calculate resistance and elastance of lung tissue strips (Leite-Júnior et al., 2003). For oscillatory mechanics, subpleural parenchyma strips of the lower lobes are cut and the resting length (Lr) and wet weight (W0) of each strip are measured (Aristoteles et al., 2013; Righetti et al., 2014). Lung tissue strips are composed of 86–90% alveoli, 5–8% blood vessels and 0.4–5% airways (Ludwig and Dallaire, 1994; Aristoteles et al., 2013). The tissue lungs are infused with Krebs solution (in mM: NaCl, 118; KCl, 4.5; NaHCO_3_, 25.5; CaCl_2_, 2.5; MgSO_4_, 1.2; KH_2_PO_4_, 1.2; glucose 10) and metal clips are glued to either end of the tissue strips with cyanoacrylate. Steel wires are attached to the clips; one side is connected to a force transducer and the other side is connected to a servo-controlled lever arm. The lever arm is capable of peak-to-peak length excursions. It is connected to a function generator, which controlled the frequency, amplitude, and waveform of the device oscillation (Ebihara et al., 2000; Pigati et al., 2015). The resting tension (T) is set by the movement of a screw thumb wheel system, which effected slow vertical displacements of the force transducer. Length and force signals are converted from analog to digital with an analog-to-digital converter and recorded by a compatible computer. The resistance (R) and elastance (E) of the lung tissue strip are estimate by the recursive least-squares algorithm to the equation of motion (Ludwig and Dallaire, 1994; Aristoteles et al., 2013; Righetti et al., 2014; Pigati et al., 2015).

T = EΔ1 + R (Δ1Δt) + K

Where T is tension, l is length, Δl/Δt is the length change per unit of time, and K is a constant reflecting resting tension. The unstressed cross-sectional area (*A*0) of the strip was obtained from the formula:

A0 (cm2) = W0(p x Lr)

In this sense, Nakashima et al. (Nakashima et al., 2008) evaluated the active process of lung immune unresponsiveness with oral ingestion of ovalbumin (oral tolerance) in guinea pigs sensitized with ovalbumin and Starling (Starling et al., 2009) evaluated the use of nitric oxide synthetase inhibitor in an experimental model of asthma. In both studies oscillatory mechanics identified the improvement in lung tissue resistance and elastance parameters as a result of treatment. Furthermore, changes in lung resistance and elastance also correlate with changes in inflammation, oxidative stress, and remodelling in the lung parenchyma (Righetti et al., 2014; Pigati et al., 2015). Pigati et al. 2015 showed similar changes in resistance and elastance of the respiratory system and lung tissue strip. Moreover, tissue strip oscillatory mechanics can also include dose-response curves after challenges with antigen or methacholine. (Lanças et al., 2006). In recent decades, several studies used this technique for measuring lung tissue resistance and elastance *in vitro* (Xisto et al., 2005; Santos et al., 2008; Starling et al., 2009; Aristoteles et al., 2013; Righetti et al., 2014; Pigati et al., 2015) and these studies helped to support the importance of alterations in the lung parenchyma of asthmatic patients (Tulić and Hamid, 2003; Mauad et al., 2004; Martin, 2008).

Lung mechanics produces a simple description of the function of the lungs and can be used to detect diseases and to analyse the effects of potential treatments. The new methodologies (lung strip mechanics and oscillatory mechanics) described above allowed a more complex and accurate description of lung function (Bates and Irvin, 2003; Leite-Júnior et al., 2003; Lanças et al., 2006). In the decades to come, lung mechanics will continue to play a fundamental role in respiratory research and clinical care.

## Lung Physiology in Human Subjects: Mechanical Ventilation and Lung Assessment

### Lung Physiology and Evolution of Mechanical Ventilators

Besides allowing mechanistic investigation on several lung diseases, respiratory physiology largely contributed to evolution of mechanical ventilation. In the past century, mechanical ventilation evolved from bulky and cumbersome negative pressure chambers (iron lungs) to modern positive pressure ventilators (Kacmarek, 2011). Over decades, many features and ventilatory modes were added to ventilators. This process was greatly assisted by knowledge gained in basic lung physiology.

Incorporation of positive end expiratory pressure (PEEP) to mechanical ventilators became widespread after the description of Acute Respiratory Distress Syndrome (ARDS) in 1967 (Ashbaugh et al., 1967) and observations of hypoxemia improvement with the use of PEEP (Ashbaugh et al., 1969). Measurements of lung compliance using equation of motion and responses to PEEP were important for defining the new syndrome. Although compliance is not part of current ARDS definition (Ranieri et al., 2012), it has been used in initial characterizations of the syndrome (Ashbaugh et al., 1967; Murray et al., 1988). Later, investigations on the role of PEEP and tidal volume in ARDS contributed to understanding Ventilation Induced Lung Injury (VILI) (Parker et al., 1990a; Corbridge et al., 1990; Dreyfuss and Saumon, 1993); and were the basis for developing Lung Protective Ventilation (Amato et al., 1998; The Acute Respiratory Distress Syndrome Network, 2000).

Some ventilatory modes added in time to ventilators were largely based on lung physiology. In Proportional Assist Ventilation (PAV), lung compliance and resistance are used to determine the amount of airway pressure delivered by the ventilator (Younes, 1992). In Neurally-Adjusted Ventilatory Assist (NAVA), electrical impulses generated by depolarization of diaphragm fibers are captured and control the level of ventilatory support (Sinderby et al., 1999). In Automatic Tube Compensation (ATC), mechanical ventilators provide additional pressure proportional to flow and endotracheal tube resistance (Guttmann et al., 1993; L’Her, 2012).

### Respiratory Mechanics Applied to Clinical Care

Assessment of lung mechanics is used in daily clinical care. In patients receiving invasive mechanical ventilation, the equation of motion is used to describe lung mechanics and to assist in the characterization of respiratory failure (Pham et al., 2017). Physicians can measure increases in resistance (Figure 2) in respiratory failure in patients with obstructive disease. Changes in resistance over the course of treatment can indicate worsening or improvement of disease status and guide ventilatory support.

In ARDS, measurements of lung compliance (Figure 2) inform the clinician about disease severity (Morales-Quinteros et al., 2019; Panwar et al., 2020; Boscolo et al., 2021). In COVID pandemic, lung compliance was employed to describe specific phenotypes: patients with hypoxemia but little amount of lung collapse and high compliance versus very low compliant lungs with large amount of lung collapse (Gattinoni et al., 2020b). The authors suggested different phenotypes could benefit from different ventilatory strategies (Gattinoni et al., 2020a). This approach was disputed by other researchers, who demonstrated respiratory compliance in COVID patients were similar to values previously reported in ARDS (Tobin, 2020; Ziehr et al., 2020; Sjoding et al., 2021) and treatment should not be changed. Early findings during a pandemic should be carefully evaluated before changing current practice (Meyer et al., 2021). Despite the controversy, use of physiological parameters to further classify ARDS patients highlights the heterogeneity of the disease (Khan et al., 2021). In the future, we might be able to learn what parameters could determine changes in treatment strategies.

Over decades, different physiological approaches have been proposed to properly set PEEP levels in ARDS patients. Obtaining lung pressure-volume curve and selecting PEEP levels according to best compliance was initially used by Amato (Amato et al., 1998). This is rather laborious and measuring lung compliance at different PEEP levels after lung recruitment could be a suitable simplification of this method. Calculating dead space and shunt fraction was also used for PEEP selection (Ferluga et al., 2018; Karbing et al., 2020; Tusman et al., 2020). Recently, measuring potential recruitment has also been proposed (Chen et al., 2020). And Talmor et al. (2008) proposed setting PEEP levels to maintain a positive transpulmonary pressure, measured using an esophageal catheter. They have shown improvements in oxygenation but failed to demonstrate decrease in mortality with this technique (Beitler et al., 2019). Unfortunately, an ideal method for PEEP selection has not been found. Some authors have used a FiO_2_-based table to guide PEEP setting. This method can be suitable in the busy ICU environment but did not reduce ARDS mortality (Brower et al., 2004). Additionally, setting PEEP based on FiO_2_ level ignores that patients might respond very differently. Physiological approaches could still be useful in addressing this problem.

**FIGURE 3:**
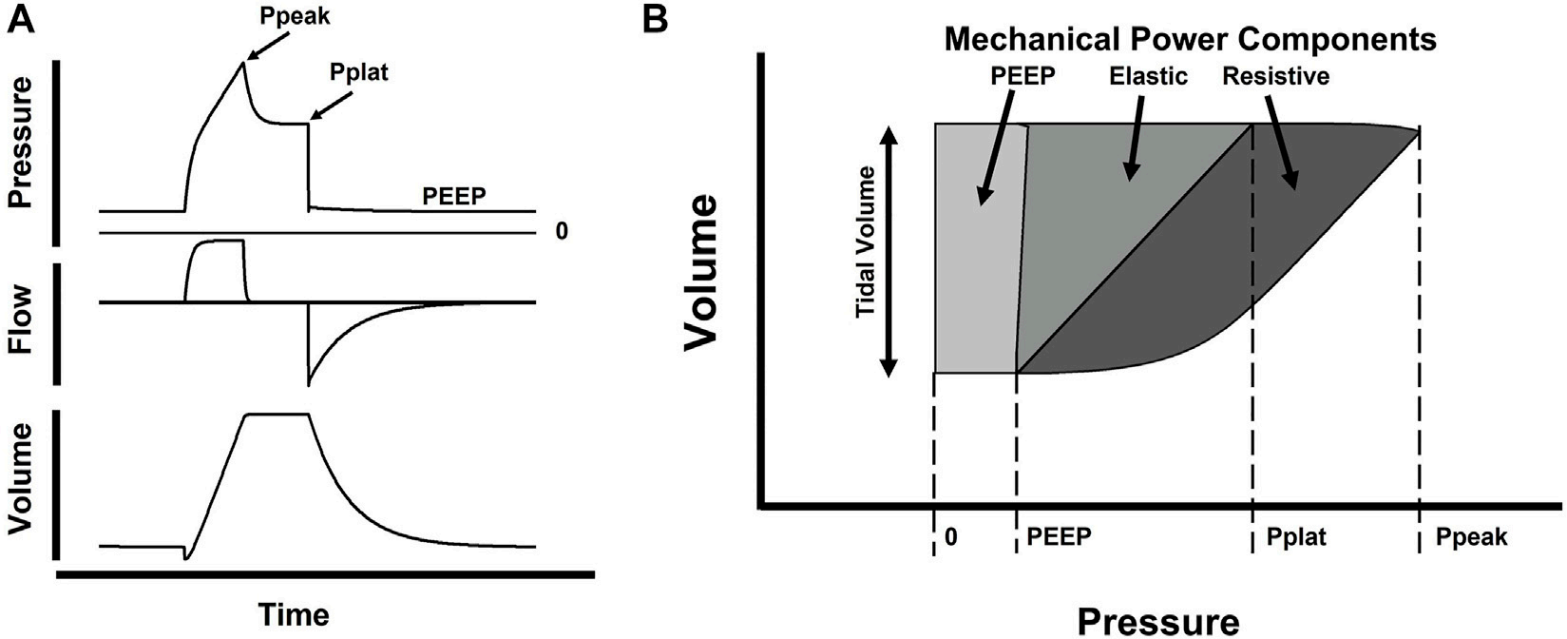
Schematic representation of Mechanical Power. In panel **(A)** we display a single breath of a patient ventilated with Volume Assist Control. Graphs show changes in Pressure, Flow and Volume over time and highlight points of interest: PEEP, peak pressure (Ppeak) and plateau pressure (Pplat, at end of inspiratory pause). In figure **(B)**, we display a Volume-Pressure loop with the same points of interest observed in panel **(A)**. Dark gray area represents Resistive Mechanical Power, change in pressure to overcome resistive respiratory forces integrated over change in volume (tidal volume); middle gray area represents Elastic mechanical power, change in pressure to overcome elastic respiratory forces integrated over change in volume; light gray area represents PEEP mechanical power, a static component of pressure representing baseline tension on the respiratory system also integrated over change in tidal volume. (Adapted from Silva et al., 2019).

More recently, Gattinoni proposed the use of Mechanical power, a new measurement of stress applied to lungs based on energy delivered during mechanical ventilation (Gattinoni et al., 2016). Mechanical power was based on equation of motion and incorporated concepts of mechanical work displayed on Campbell’s diagram (Cabello and Mancebo, 2006). Each component of the equation of motion was multiplied by the change in volume and Respiratory rate (Figure 3) to evaluate the individual contribution to lung injury (Silva et al., 2019). The usefulness of this new analysis is still under investigation. Some authors believe it does not add substantial new information to mechanical ventilation management (Costa et al., 2021). On the other hand, mechanical power can be associated to biomarkers of lung deterioration (Rocco et al., 2020). At a minimum, it underscored the importance of respiratory rate as a source of stress to lungs during ventilation.

The assessment of respiratory mechanics in spontaneously breathing patients has always been a challenge. Respiratory effort should be accounted for when applying equation of motion (Grinnan and Truwit, 2005). Esophageal catheters can be used to measure patients’ effort (Baydur et al., 1982), allowing the use of equation of motion even in non-paralyzed patients (Figure 2). On the other hand, new lung mechanics techniques evolving from experimental physiology can be used in spontaneously ventilated patients. Notably, forced oscillation technique, or oscillometry, has contributed to a better understanding of lung function in clinical routine and in research, particularly in patients with obstructive disease (Peterson-Carmichael et al., 2016; Hantos, 2021; Lundblad and Robichaud, 2021).

### Lung Protective Ventilation. Tidal Volume Challenge

Initial basic research on the effects of PEEP and tidal volume in ARDS (Parker et al., 1990b; Carlton et al., 1990; Corbridge et al., 1990; Dreyfuss and Saumon, 1993) later translated in trials investigating mechanical ventilation management strategies (Amato et al., 1998; The Acute Respiratory Distress Syndrome Network, 2000). Current guidelines for ARDS management suggest limiting tidal volume to 6 ml/kg and plateau pressure to 30 cm H_2_O (Griffiths et al., 2019; Papazian et al., 2019). This strategy was named lung protective ventilation. It revealed that mechanical ventilation can be harmful to lungs and defined new targets for the health team. It also introduced the challenging idea that aiming normal blood gas values during mechanical ventilation could lead to worse outcomes. Higher pCO_2_ levels (permissive hypercapnia) generated by reduction tidal volume were initially regarded as benign. Later, some authors recognized hypercapnia was associated to impaired immunologic response and vascular tonus dysfunction. Hypercapnia increases pulmonary resistance and can contribute to acute cor pulmonale (Repessé and Vieillard-Baron, 2017; Barnes et al., 2018). Nevertheless, the best current evidence still recommends limiting tidal volume for ARDS patients despite CO_2_ elevation. Patients with hypercapnia in this setting could be managed with additional rescue strategies (Repessé and Vieillard-Baron, 2017).

Lung protective ventilation concepts were further explored in a later reanalysis of the initial ARDS trials (Amato et al., 2015). The authors used a multilevel mediation analysis to conclude that driving pressure (plateau pressure—PEEP) was the variable most significantly associated to mortality. Although ARDS guidelines advised against the use of targets of driving pressure (Papazian et al., 2019), this measurement integrates the concepts of disease severity (lung compliance) and ventilator management (tidal volume). Further prospective trials are required but driving pressure could become an essential parameter to monitor in the future.

The success of managing mechanical ventilation during ARDS led several authors to suggest the use of lung protective ventilation in all patients receiving mechanical ventilation. Indeed, initial observational studies suggested better outcomes or decreased inflammatory cytokine production when limiting tidal volume in non-ARDS patients (Determann et al., 2010; Neto et al., 2012; Fuller et al., 2013). The initial observations were not confirmed in a properly conducted clinical trial (Simonis et al., 2018). As surprising this might look, one must remember that patients with ARDS have lungs very different from normal and from other patients on mechanical ventilation. Limiting tidal volume can be useful in some scenarios, as operating rooms, but can be very challenging in ICU. Some individuals would require deep sedation and muscle paralysis to limit tidal volume regardless of the ventilatory mode selected, adding significant morbidity to patient care. Patients waking from sedation after uneventful surgery that develop large tidal volume on spontaneous breathing should probably be extubated and not deeply sedated. COPD patients could benefit from lowering tidal volume and minute ventilation, but probably would handle very poorly high respiratory rates that might be required if tidal volumes are greatly reduced (Marini, 2011).

Even in ARDS patients, duration of strict protective ventilation can bring challenges to caring team. Although essential in the beginning of care, limiting tidal volumes can be difficult in some patients once sedation and muscle paralysis are withdrawn. Maintaining long periods of sedation and paralysis can lead to muscle weakening and prolong time on mechanical ventilation (Kress et al., 2000; Girard et al., 2008; Reade and Finfer, 2014). On the other hand, patients with high ventilatory drive will produce large tidal volumes regardless of ventilatory mode selected (Yoshida et al., 2013; Papazian et al., 2019).

The importance of ventilatory drive and its contribution to lung injury is still under investigation. It has been recognized that patients can generate significant amount of inspiratory pressure during respiratory failure, both before and after being intubated. Large inspiratory pressure swings will translate into large transpulmonary pressure irrespective of the settings on non-invasive ventilation device (before intubation) or mechanical ventilator (after intubation) (Brochard et al., 2017). Even when volume assist control mode is selected, patients with large swings generate intrathoracic pressure reduction that can produce lung edema, increases in left ventricle afterload and double triggering in ventilator, which doubles or triples tidal volumes (Pohlman et al., 2008; Pinsky, 2018; Sottile et al., 2020). The contribution or spontaneous breathing patterns to lung injury has been named P-SILI (Patient Self Inflicted Lung Injury). Although some authors have advised against using those new concepts to manage ventilator at this stage due to lack of experimental and clinical data (Tobin et al., 2020), this will be an important topic to explore. The relative importance of spontaneous effort in producing lung injury will need to be balanced against the deleterious effects of extending sedation and muscle paralysis.

Guidelines for properly managing those patients are still missing. We will need further cooperation between basic and clinical science to understand the limits of lung protective ventilation. We need to understand when this strategy is absolutely required warranting muscle paralysis; and when we can be more flexible on these rules. We also need to understand what other lung disorders require limiting tidal volume.

### Respiratory Mechanics in Obstructive Lung Diseases

Patients with respiratory failure secondary to obstructive lung disease can require mechanical ventilation. Ventilatory strategies for these patients are designed to avoid air trapping and intrinsic PEEP (PEEPi) generation (Reddy and Guntupalli, 2007). PEEPi is produced when expiratory time is insufficient to allow complete tidal volume exhalation, usually when both airway resistance and respiratory rate increase. Air is trapped inside the lungs, increasing intrathoracic pressure, decreasing venous return and increasing patients’ effort to trigger the ventilator and generate inspiratory flows (Pepe and Marini, 1982; Marini, 2011). Mechanical ventilator settings should allow enough expiratory time to minimize the impact of PEEPi. Although limiting tidal volume can be beneficial, low minute ventilation and high expiratory time should be main targets (Reddy and Guntupalli, 2007).

In obstructive patients, equation of motion can be used to measure lung resistance and monitor the response to treatments: bronchodilators, antibiotics and management of airway secretion. PEEPi can be measured with expiratory pause, informing bedside decisions on setting ventilator parameters.

However, there are limitations for the use of lung mechanics in those patients. Equation of motion is usually applied only to inspiratory phase, where airway pressure variation is significant. Measurement of airway resistance during expiration is often neglected despite its importance in generating PEEPi. Some patients in respiratory failure reach a condition of expiratory flow limitation, where airways collapse at the mid-end expiration (Bates, 2009). In this scenario, expiratory flow becomes independent of pressure gradient and expiratory airway resistance cannot be defined using equation of motion.

Additionally, patients on mechanical ventilators are often awake and have spontaneous breathing. In this setting, if pressure generated by respiratory muscles is not measured (Figure 2B), lung mechanics cannot be properly assessed. PEEPi measurement requires long expiratory pause which is usually not possible in patients with spontaneous breathing (Grinnan and Truwit, 2005). And since PEEPi is highly dependent on respiratory rate, values of PEEPi obtained during neuromuscular block are hardly valid when patients resume respiratory effort.

In the future, widespread use of esophageal pressure catheters can allow monitoring of respiratory effort and assessment of lung mechanics in patients with spontaneous breathing (Grinnan and Truwit, 2005). PEEPi can also be measured using esophageal pressure monitoring (Marini, 2011). Methods for measuring expiratory resistance and detecting expiratory flow limitation can become easier. Finally, oscillatory mechanics at the bedside can provide information on dissipative forces of the respiratory system even in patients with spontaneous breathing (see section Oscillometry). It could then allow the analysis of lung resistance in patients with obstructive lung disease without the requirement of deep sedation.

## Lung Physiology Still Not at Bedside Primetime

For different reasons, several physiological approaches developed in the lab did not reach bedside yet and will be discussed in the paragraphs below. Some of these techniques are laborious or provide data clinicians are not ready to use. Some techniques, on the other hand, did become commercially available but are still seldom employed.

Multiple inert gas elimination technique (MIGET) was designed for determining ventilation/perfusion distribution throughout the lungs using several gases with different solubility on blood. MIGET advanced knowledge of respiratory physiology in different species and elucidated mechanisms of hypoxia in different disorders (Wagner, 2008). The technique is very laborious and requires not only injection of several gases but also a pulmonary catheter for measuring cardiac output and gas detector. It provides useful clinical information and could be used to select PEEP levels or describe functional lung behaviour during treatment. Its complexity, however, prevented widespread clinical application.

Electrical Impedance Tomography (EIT) evolved from very simple and inaccurate devices to monitor respiratory rate into complex continuous monitors of lung ventilation. Recent advances in the technique also included perfusion and V/Q distribution measurement using electrocardiography-gated impedance signals or following hypertonic saline infusion (Costa et al., 2009; Nguyen et al., 2012). There should be some caution in interpreting perfusion measured by impedance technique. Impedance signals should not be able to detect perfusion defects in small capillaries if pulmonary blood flow to major arteries remains unchanged (Deibele et al., 2008). Therefore, some authors believe perfusion EIT will be more useful as a non-invasive tool for diagnosing pulmonary embolism rather than small V/Q mismatch (Maciejewski et al., 2021). However, the technology is still evolving and methods for diagnosing small perfusion defects could arise.

Although not used in many ICUs, EIT became commercially available and can provide useful information on ventilation during patient care. It can be used to improve PEEP titration since it displays both overdistention and lung collapse. It also provides a visual and numerical analysis of ventilation homogeneity, can detect pneumothorax in real time and displays patterns of ventilation during spontaneous efforts (Coppadoro et al., 2020; Maciejewski et al., 2021). In the present, there is not enough supporting evidence for the use of EIT, but it can become an important monitoring device in the future.

Measuring resting lung volumes has always been difficult in clinical and research settings. Nitrogen-washout measurements have been proposed by some authors. The technique uses changes in FiO_2_ concentrations and nitrogen dilution to estimate lung volumes (Olegård et al., 2005; Dellamonica et al., 2011). The process, however, is time-consuming and cannot be applied continuously in a busy ICU. Nevertheless, measuring resting lung volumes can be used to properly set tidal volumes. Guidelines for ARDS management suggest limiting tidal volumes to 6 ml/kg of ideal body weight, which is calculated based on patient height to correct for different lung sizes (Papazian et al., 2019). However, ARDS patients have different lung volumes not only because of different body constitution but also because of extension of the disease (Chiumello et al., 2008; Mattingley et al., 2011). Scaling tidal volume to actual size of lungs could allow settings targeted not only to each patient, but also to each phase of the disease (Chiumello et al., 2008; Macintyre, 2016; Umbrello et al., 2017; Pelosi et al., 2021).

Patients’ effort monitoring has been possible to measure for decades using esophageal catheters (Baydur et al., 1982). Although commercially available and proposed by many different research groups, the technique is seldom employed (Akoumianaki et al., 2014). Misplacement of the catheter, patients’ discomfort and lack of clear clinical benefit could explain the low enthusiasm for the approach. On the other hand, as we manage patients with less sedation and greater levels of interaction with ventilators, measuring effort can become important. If future research relates respiratory effort to lung injury, spontaneous breathing should be carefully handled.

### Oscillometry

Oscillometry or forced oscillation technique consists of applying flow or pressure oscillations at the entrance of the airways and monitoring the response obtained with the oscillations in order to calculate the impedance of the respiratory system. Impedance is the mechanical load of the respiratory system to ventilation.

Initially, the forced oscillation technique was used in apnea situations so that voluntary respiratory efforts did not mask the actual physiological condition of the respiratory system. However, with signal processing techniques, it became possible to use oscillometry in spontaneously ventilating patients by superimposing a high-frequency pressure waveform on the tidal breathing pattern (Peterson-Carmichael et al., 2016) and still calculate the impedance of the respiratory system with the effects of breathing minimized.

The constant-phase model (see section ‘Respiratory mechanics in animal models’ and Figure 1) applied in small rodents is not suitable for patients because of the low frequency broadband excitation needed (Lundblad and Robichaud, 2021). So, the analysis of the human respiratory impedance is based mostly on the frequency response behaviour of impedance, a complex mathematical function with real and imaginary components. This analysis strongly helps the understanding of respiratory physiology, as the real component is related to dissipative energy (resistance) and the imaginary component is related to conservative energy (elastance) of the respiratory system (Lundblad and Robichaud, 2021).

According to Hantos (Hantos, 2021), manoeuvres using oscillometry involving large but slow changes in lung volume allows for fine mapping of respiratory mechanics exceeding the tidal range and a novel intra-breath modality is capable of tracking the dynamic changes in respiratory system.

Technical standards for respiratory oscillometry have been published (King et al., 2020) and commercial devices are becoming popular.

## Back to Bench

Basic science and lung physiology helped to develop and advance mechanical ventilation at bedside and they still can be very important for the challenges ahead.

Lung protective ventilation does not answer all questions in respiratory failure. It was crafted long ago, when mechanical ventilation care was substantially different. Awake patients interacting with ventilators bring additional challenges. How far should we go to limit tidal volume? Should we continue to keep low levels of sedation in patients with high respiratory drive? Should we tolerate higher tidal volumes once the initial inflammatory phase of ARDS is over and oxygenation starts to improve? And how should we handle patients without ARDS with high respiratory drive and tidal volumes?

Measuring effort, lung volumes, lung inhomogeneities, pattern of ventilation and V/Q distribution or mechanical power can provide some of these answers. At the same time, ventilators used for small animals incorporated some of the technologies developed at the bedside, as pressure support ventilation. P-SILI and the effects of respiratory effort can be further investigated in animal models.

The advent of ECMO (extracorporeal membrane oxygenation) has brough additional complexity to the field. Once limited to operating rooms, ECMO use in ICU became more popular after influenza (H1N1 in 2009) and COVID (2020) pandemics (Combes et al., 2018; Barbaro et al., 2020). Although very expensive and invasive, ECMO can provide all the respiratory support required by some patients. Ventilators can then be adjusted to provide very minimum ventilation. The optimal setting and how long a patient should be maintained in ECMO are still under investigation (Tonna et al., 2021).

## Conclusion

The study of lung mechanics has substantially contributed to development of knowledge of respiratory diseases. It was also a cornerstone in the creation and evolution of mechanical ventilation. The combination of basic, translational and applied sciences has proved very useful in respiratory physiology leading not only to better understanding physiopathology but also to designing supportive treatment. In the years to come, we expect this partnership to continue as we face new challenges in managing patients with respiratory failure.
